# Relationship between insulin administration and substrates oxidation during continuous aerobic exercise in adolescents with type 1 diabetes mellitus

**DOI:** 10.1186/1758-5996-7-S1-A235

**Published:** 2015-11-11

**Authors:** Juliana Pereira Decimo, Luís Paulo Gomes Mascarenhas, Valderi de Abreu de Lima, Camilla Kapp Fritz, Andréia Araujo Porchat de Leão, Neiva Leite, Luiz de Lacerda Filho, Suzana Nesi França

**Affiliations:** 1 Universidade Federal do Paraná, Pinhais, Brazil

## Background

The risk of hypoglycemia during and after exercise is a limitation to regular practice. In type 1 diabetes patients.

## Objective

To compare the influence of different intervals of insulin administration, to identify the proportion of oxidized energy substrates at different times of continuous aerobic exercise and evaluate the relationship between oxygen uptake and glycemic variation at the end of the exercise.

## Materials and methods

Six patients with type 1 diabetes mellitus were evaluated: 6 pubertal, BMI Z-score 0.56±0.48; chronological age 13.7±2.6y; duration of disease 6.5±4.26y; mean HbA1c 9.13±1.7%. Patients took breakfast and insulin at the clinic (mean basal insulin 0.47±0.07U/kg/d and fast insulin 0.33±0.12U/kg/d). Continuous ergometric test at moderated intensity (60% VO2max) was performed for 30 min, one and two h after insulin (T1 and T2). Intensity was controlled through oxidation and heart rate measured at 10 and 30 min of exercise with K4b2® gas analyzer. Blood glucose levels were measured at the beginning, 10 and 30 min.

## Results

**Figure 1:**
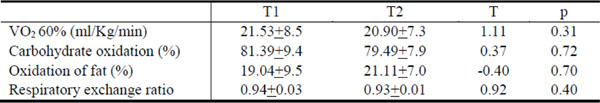
Oxidation of substrates 1h and 2h after insulin administration.

**Figure 2:**
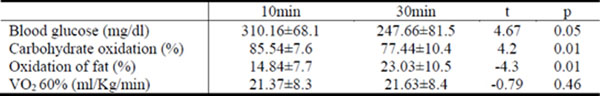
Oxidation of substrates at 10 and 30 min of exercise.

Initial blood glucose was 299±48mg/dL, VO2max 40.7±9.63ml/Kg/min. No difference was found between intervals of insulin administration regarding the oxidation of substrates (Figure 1). Difference between 10 and 30 min of exercise are shown in Figure 2. The glycemic variation (VG) was -65.41 mg/dL. Regression analysis showed correlation (r=0.98) between glycemic variation, fast insulin and VO2max with a R2=0.977, predictive equation: VG=347.108- (5.269 * VO2max) – (579.49*fast insulin).

## Conclusion

Substrate oxidation wasn't influenced by insulin administration before exercise. A higher carbohydrate oxidation was observed at 10min of exercise and subsequent increase in fat oxidation at 30min. Fast insulin dosis and VO2max are a good predictor of glycemic variation at the end of continuous moderate aerobic exercise.

